# Methods for Inferring Health-Related Social Networks among Coworkers from Online Communication Patterns

**DOI:** 10.1371/journal.pone.0055234

**Published:** 2013-02-13

**Authors:** Luke J. Matthews, Peter DeWan, Elizabeth Y. Rula

**Affiliations:** 1 Activate Networks Inc., Newton, Massachusetts, United States of America; 2 Healthways Center for Health Research, Healthways Inc., Franklin, Tennessee, United States of America; Umeå University, Sweden

## Abstract

Studies of social networks, mapped using self-reported contacts, have demonstrated the strong influence of social connections on the propensity for individuals to adopt or maintain healthy behaviors and on their likelihood to adopt health risks such as obesity. Social network analysis may prove useful for businesses and organizations that wish to improve the health of their populations by identifying key network positions. Health traits have been shown to correlate across friendship ties, but evaluating network effects in large coworker populations presents the challenge of obtaining sufficiently comprehensive network data. The purpose of this study was to evaluate methods for using online communication data to generate comprehensive network maps that reproduce the health-associated properties of an offline social network. In this study, we examined three techniques for inferring social relationships from email traffic data in an employee population using thresholds based on: (1) the absolute number of emails exchanged, (2) logistic regression probability of an offline relationship, and (3) the highest ranked email exchange partners. As a model of the offline social network in the same population, a network map was created using social ties reported in a survey instrument. The email networks were evaluated based on the proportion of survey ties captured, comparisons of common network metrics, and autocorrelation of body mass index (BMI) across social ties. Results demonstrated that logistic regression predicted the greatest proportion of offline social ties, thresholding on number of emails exchanged produced the best match to offline network metrics, and ranked email partners demonstrated the strongest autocorrelation of BMI. Since each method had unique strengths, researchers should choose a method based on the aspects of offline behavior of interest. Ranked email partners may be particularly useful for purposes related to health traits in a social network.

## Introduction

Social network analysis has elucidated how the health of individuals is strongly interconnected with the health of their social ties. Much of the recent work in this area was spearheaded by Christakis and Fowler, researchers who used self-reported contacts to map the social network of participants in the Framingham Heart Study. In a series of articles, researchers demonstrated that changes in health traits like smoking, obesity, and depression correlated across the ties of this network [1]–[4]. A subsequent study that focused on an online therapeutic network, participants in the internet smoking cessation program QuitNet, demonstrated that this online community shared many of the properties of other social networks [5]. Still other studies have shown that intelligent structuring of online communities produced enhanced participation in health programs [6], [7].

No research to date, however, has demonstrated that health traits correlate across social ties among coworkers. Although Christakis and Fowler [2] showed that coworkers at small companies (those with <6 individuals) appeared to quit smoking together, no one has assessed how patterns of social ties within a larger company may be identified to evaluate correlations of health traits across network connections. The network effects of friendship shown in the Framingham studies may not replicate in coworker settings because the Framingham participants each named only a few close friends from among all their social contacts [1]–[4]. If network health effects occur in employee populations, then mapping these networks may provide substantial new avenues for employers to improve the health of their employees, resulting in a healthier, happier, more productive, and less costly workforce.

A key methodological barrier to applying social network analysis to the health of employee populations is obtaining a sufficiently comprehensive source of network information. Survey-based methods are the validated approach for mapping of health-relevant social networks, but surveys can be slow to conduct and often suffer from low completion rates. Incomplete data produce networks with missing social ties, which can confound network-based inferences and applications [8].

We turned to email data as a potential source of network information that would be complete and readily available for most major employee populations. If networks derived from emails can be used to predict survey-based ties, and be shown to meaningfully predict important health traits, then this would open up the use of email networks in many applied health settings. Although email may primarily represent professional communication, it is plausible that individuals who are closely connected professionally influence one another more broadly. A primary purpose of this study was to test this hypothesis by testing alternative means of email-network generation and investigating how well these networks predict BMI.

We assessed the validity of several methods for inferring health-relevant coworker social networks from email data. We used a network derived from a Name Generator Survey that collected self-reported social ties relevant to one’s health behaviors to optimize three qualitatively distinct methods to construct social networks from email data. We then assessed how an important health trait, Body Mass Index (BMI), covaried across social ties in each of the networks. We also examined the correlations of many common network metrics across the Name Generator and email networks.

### Constructing Social Networks from Email Data

Email communication may be particularly useful for inferring ‘offline’ social relationships because email requires some nominal investment of time to maintain an online relationship. In this manner email differs substantially from online forums like Facebook and Twitter, in which a participant can acquire dozens of friends or follow multiple Twitter feeds with only a few minutes of time.

Email is, furthermore, one of the primary means of communication within businesses, and frequently is available as a data source at least to businesses internally. Networks generated from email may represent a fruitful tool for many applications of social network analysis to the internal dynamics of businesses. We recognize that there may be other equally valuable sources of network information in other types of media for internal business communications, such as phone logs and electronic instant messages. Email is a major mode of communication for many employers including Healthways, the subject of this particular case study, and so was a logical focus of this investigation. Additional work would be required to generalize the results below to other communication media, although we see no reason *a priori* why other forms of communications, particular electronic textual communication, would differ fundamentally from email with respect to their correspondence to offline social relationships. In this study, we applied three qualitatively distinct techniques for inferring unweighted social relationships from email data and evaluated the performance of each technique by reference to a social network from a survey instrument. Several previous studies have constructed social networks from emails using mostly ad hoc threshold criteria for designating a social tie from email communication logs [9], [10]. Some approaches have used a single *a priori* threshold of email frequency that constituted a social tie [9]–[12], while other approaches used weighted ties based on the number of emails sent and/or received [11]. Few studies have systematically examined the performance of qualitatively distinct methods for processing email against a reference network from a survey tool [13] and this is the first time that network correlation of a health trait has been assessed across networks generated from email data.

Drawing from previous studies, we inferred social networks using a single threshold on emails sent/received. We also assessed the performance of two novel methods for construction of networks from email data (detailed below in Methods). One method used a logistic regression model on nine email characteristics to predict the probability of an offline network tie. Another method ranked email contacts by number of emails exchanged for each individual and created thresholds of email rankings. We developed criteria for inferring offline social ties by referencing a network map developed using data from a ‘Name Generator’ survey that was completed by employees who also appeared in the email logs. The Name Generator Survey collected information on coworker relationships by prompting them with a question asking with whom at the workplace they would engage in particular discussions or activities.

### Critiquing the Usefulness of Networks Constructed from Email

The value of email-based networks depends on the desired goal [11], [12]; for example, whether a researcher desires to predict a particular trait that correlates across network ties or to identify central individuals. We addressed the usefulness of the email networks for each of these purposes, with particular attention to the usefulness of email networks for predicting important health traits.

To determine which email network best predicted individuals’ traits, we assessed the correlation of Body Mass Index (BMI) across ties in the email networks. Previous research indicates that BMI correlates across traditional network ties generated by survey that asked about friendships from among all of their social contacts [1]. Additionally, we chose to study BMI because it is altered by many physiological, behavioral, and environmental factors that characterize individuals [14], [15]; therefore, a network’s ability to predict BMI may indicate its general utility to predict aspects of an individual’s health and lifestyle.

Second, we examined how common network measures of degree, betweenness, closeness, eigen vector centrality, and transitivity compared across the networks. Each of these measures, defined in Methods, describe different aspects of an individual’s position within the overall social network structure [16]. They are employed for diverse purposes in academic and applied network research. Our goal was to assess which methods of inferring networks from email data obtained network metrics that were most similar to those identified in the offline social network mapped using Name Generator Survey responses.

The results from each of these analyses were intended to provide a comprehensive assessment of each of the methods for using email data to create social networks, how well these mapped networks replicated the offline network generated using survey data, and the extent to which BMI correlated across network ties.

## Methods

### Ethics Statement

This study was not submitted to an institutional review board (IRB) because only de-identified survey data and existing, de-identified email and BMI data were used for this analysis in alignment with IRB exclusion criteria outlined in the Code of Federal Regulations (Code of Federal Regulations. Protection of Human Subjects. Vol 45 C.F.R. § 46.101 2005). No IRB waived the requirement of formal ethical approval; the Code of Federal Regulations states clearly why this research is exempt.

(b) Unless otherwise required by department or agency heads, research activities in which the only involvement of human subjects will be in one or more of the following categories are exempt from this policy:

(4) Research involving the collection or study of existing data, documents, records, pathological specimens, or diagnostic specimens, if these sources are publicly available or if the information is recorded by the investigator in such a manner that subjects cannot be identified, directly or through identifiers linked to the subjects.

Code of Federal Regulations. Protection of Human Subjects. Vol. 45 C.F.R. § 46.101 2005

Participants in this study provided written informed consent prior to participating in the annual biometric screenings conducted by their employer (Healthways Inc.). These biometric screenings are a standard practice at Healthways.

All data used in the present study were analyzed anonymously using unique identification numbers for each employee. All data were highly secure at all times during the analysis, as even de-identified data were treated by both Healthways and ANI as Protected Health Information (PHI) and processed via a process compliant with the United States Health Insurance Portability and Accountability Act (HIPAA).

Healthways collected all data used in this study (BMI, email logs, and name generator surveys) from their own employees. These data were provided by Healthways to ANI for analysis.

### Description of Data Collected

We collected BMI data, and social network survey and email data from employees of Healthways, a mid-sized public healthcare company. BMI values were collected during an annual biometric screening, which is a standard practice at Healthways. During the biometric screenings, clinicians collected objective measures of height and weight for employee BMI calculation on a voluntary basis.

Offline social ties were inferred from information collected from a Name Generator Survey that asked each participant four questions (Table 1). Participants could name up to 5 people in response to any question, for a potential total of 20 named individuals. We considered any named individual to share an undirected network tie with the survey respondent. Most Healthways employees completed the survey in the online Survey Monkey application (SurveyMonkey: Free online survey software & questionnaire tool, http://www.surveymonkey.com, last accessed Dec 14, 2011) and were offered as an incentive the chance to win any of a number of small prizes of nominal value as well as a chance to win one iPad. Prior to the Survey Monkey, participants also had a chance to fill out a paper version of the Name Generator. The paper survey was administered in January 2011, while the Survey Monkey took place from May 4–31, 2011. Both surveys were voluntary and informed participants of the intended use of the data to map the social network of the organization. Prior research on name generator surveys indicates artifacts can occur from the way the survey is administered, particularly in the context of interviewer effects during in person or phone surveys [17]. While such artifacts can never be eliminated from any dataset, the fact that most respondents completed the survey online and at the same time period should help to minimize such artifacts between subjects in the present study.

**Table 1:** Name generator survey questions.

Which colleagues do you spend free time with, either in the work environment or outside? (please list up to 5)
Which colleagues would you feel comfortable discussing important personal (non work) matters with? (please list up to 5)
Which colleagues would you be most likely to discuss health matters with or ask for health advice? (please list up to 5)
Which colleagues would you be most likely to engage in an outside activity with, such as a walk, playing a sport or a game, volunteering, or taking a course?

Healthways provided monthly email data files to Activate Networks for analysis and processing. These email files included time and date stamped records of all emails sent to and from Healthways email accounts. Each email account was given a unique identifying number, in lieu of employee name, to protect individual privacy. Emails sent to or from a source outside of Healthways had a null value for the ID number and thus were easily excluded from the dataset. We also excluded pairs of individuals from the email data if neither the sender nor the receiver responded to or was named in the Name Generator Survey. We used email records for a five month period from January 2011 through May 2011.

### Optimizing Email Networks to the Name Generator Survey

#### Thresholding on the number of emails sent and received (The single recipient network)

Several authors have proposed that constructing social networks from email data that are relevant to offline behavior involves deciding upon a suitable threshold of emails sent and/or received that is most predictive of an offline social tie [9]–[11]. We tested one implementation of this concept by calculating the sum of all single-recipient emails between each pair of individuals over the study period. We then constructed threshold cutoffs for social ties at thresholds of single-recipient email volume from an average of 1 email per month up to 20 emails per week. For each threshold cutoff, we postulated a social tie between individuals with values above the threshold and an absence of tie for values below the threshold. We calculated the correlation of the resultant adjacency matrix for each email network to the adjacency matrix of the offline network, mapped using Name Generator results. An adjacency matrix is an N by N matrix where N is the total number of individuals in the population and each cell in the matrix thus represents a single pair of individuals in every possible combination. The value in each cell is a 0 for pairs that lack a social tie and a 1 for individuals who have a social tie. We included individuals who were named in the Name Generator Survey even if they did not respond to the survey themselves. We then selected the Single Recipient Network based on the threshold value that produced the largest Pearson correlation with Name Generator ties. This method necessarily resulted in an undirected set of network ties being inferred from the email data. Undirected networks exhibit only bi-directional social ties; that is, if A is tied to B then B is tied to A. Directed networks do not have this property such that A could be socially tied to B without B having a reciprocal tie to A.

#### Thresholding on logistic regression predicted probabilities (The logistic regression network)

As another means for inferring offline social ties from email data, we constructed a logistic regression model from the email parameters in Table 2. We scored whether or not a Name Generator tie occurred between each pair of individuals in the email data and used this vector as the dependent variable. We estimated the logistic regression model via maximum likelihood, and then used the fitted model to predict the probability of a tie for each pair [18]. In estimating this model, we first eliminated all pairs of individuals if they never exchanged any form of email. We quantified the number of emails sent relative to an individuals’ emailing frequency by calculating z-scores for the email senders. We observed that the distribution of total emails sent by individuals was highly skewed with an elongated upper tail. The number of emails to particular recipients by particular senders was also skewed. To accommodate this skew, we log-transformed the number of emails sent prior to z-score calculation. We also calculated an email *asymmetry index* for each pair of individuals. We did this because we thought the evenness of an email communication may be predictive of a stronger social tie; thus, by including an asymmetry index in the model we could adjust the predicted tie probability based on the evenness of email exchange. This index was calculated as the absolute difference between one half the total number of emails exchanged by a pair and the number of emails sent by one individual to the other member of the pair. The absolute difference was then divided by one half the total number of emails exchanged by that pair. For each pair of individuals, this calculation yielded a number from 0 to 1 where 0 represented a perfectly even exchange of email and 1 represented all the email going from one individual and to the other.

**Table 2:** Parameters for the logistic regression model to predict Name Generator ties.

Independent Variable	Definition	Dummy For Zeros?*
z-score sent single	The logged single recipient emails sent by individual 1 standardized to 0 mean and standarddeviation of 1 based on all other email partners for individual 1	
z-score sent multiple	The logged multiple recipient email sent by individual 1 standardized to 0 mean and standarddeviation of 1 based on all other email partners for individual 1	
single recipient†	The number of single recipient emails between members of a pair	yes
multiple recipient†	The number of multiple recipient emails between members of a pair	yes
sum file sizes†	The sum of file size for all the emails between members of a pair	yes
shared contacts	The number of other individuals that both members of a pair have emailed	
secondary ties	The number of other individuals with whom the email sender has more than one “shared contacts”	
asymmetry single	The asymmetry index for single recipient emails	
asymmetry multiple	The asymmetry index for multiple recipient emails	

* This field indicates a dummy variable was also included. If a data point for the row variable was a 0, the dummy took on a value of 1. Otherwise the dummy was 0. Row variables with blank entries did not exhibit over-dispersion of zeros and so did not require dummy variables.

† Variable was log transformed to better meet generalized linear model assumptions.

We considered the most probable pairs (according to the model) to be social ties, and tested a series of probability thresholds to find which produced the highest correlations with the Name Generator ties. We created thresholds at units of 0.01 from a value of 0.01 up to the highest fitted probability value. We then selected the Logistic Regression Network based on the threshold value that produced the largest Pearson correlation with Name Generator ties. This method produced a directed network from the email data, because a tie could have a higher fitted probability in one direction than another. We retained this directional information in the test of BMI against the networks, but considered only undirected ties for all comparisons of egocentric network measurements. This was done because network measures would not be comparable across undirected and directed network data.

#### Thresholding on ranked email partners (The ranked partner network)

As a third method of constructing social ties from email data, for each individual we ranked the sum of single-recipient emails sent to or received from every other individual with whom they exchanged emails. The email partners of each individual were given a rank from highest (most email exchanges) to lowest (least email exchanges). We thresholded on the highest ranked emails for each individual and considered these pairs to be social ties. We varied the threshold from 2 to 25 at intervals of one rank; at each rank threshold we calculated the correlation of the resultant email network to the Name Generator Network. We then selected the Ranked Partner Network from the threshold that produced the largest Pearson correlation with Name Generator ties. As with the Logistic Regression Network, the Ranked Partner Network resulted in a directed set of network ties because an individual might rank highly among another’s email partners even if that alter is not ranked highly for the ego. As with the Logistic Regression Network, we retained this directional information in the tests with BMI but considered only undirected ties for all other statistical comparisons.

### Testing Email Networks against Body Mass Index with Network Autocorrelation

After completing three alternative constructions of social networks from email data, we compared how well each predicted BMI. For this analysis, we included individuals who appeared in the email data, the Name Generator Network, and in the biometric BMI data set. We assessed the patterning of BMI on the alternative networks with a network autocorrelation model as implemented in the R package “sna”, function “lnam” [19]. Network autocorrelation models are used to relate traits, for which each individual has only one value, to network ties, for which each individual usually has multiple values [20]–[22]. In our implementation, we predicted deviation in an individual’s BMI from the population mean based on the BMI of their immediate social ties (using a simultaneously autoregressive – SAR – model). The SAR model can be described as



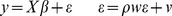



Where *y* is the dependent variable (BMI in this case), *Χ* is a matrix of predictor variables, *β* is a vector of regression coefficients of *Χ,* and *ε* is the residual variation in *y* not explained by the independent variables. In the context of network effects, *ε* is not distributed independently among the data points. Rather, tied individuals have similar deviations from their expected value. This is expressed by the parameters *w*, which is the matrix of normalized social network weights, *ρ*, the strength of network autocorrelation, and *ν*, a vector of independent residual errors.

Under any process that creates similarity among socially tied individuals, the network autocorrelation parameter, *ρ*, will vary from 0 to 1. At *ρ* = 1, an individual’s deviation from their fitted value in an ordinary regression reflects the average deviation of their social ties, while at 0 no relationship is observed between the network and the distribution of BMI values. We included only an intercept term as an independent variable; therefore, the network autocorrelation in our model expressed the network-associated deviation from the mean BMI.

Network autocorrelation methods account for the statistical nonindependence of network data by including trait values for individuals (BMI) only once in the equation and by estimating a single parameter for the overall network effect (*ρ*). The model finds the parameter value of *ρ* that maximizes the data likelihood given the observed BMI values and the observed network ties. This method does not distinguish whether the network autocorrelation results from confounding environmental features, homophily (the tendency of those with similar traits to form social ties), or social influence. Network autocorrelation can model any of these processes, which suited our goals for this project as we sought to assess how well different methods for constructing an email network would produce a network capable of predicting an important health trait. We were not trying to determine the underlying causes of network autocorrelation in the health trait itself.

We row-standardized the social network adjacency matrices to obtain the expected correlations for each pair of individuals [23]. This assumes that all individuals experience a similar level of autocorrelation, but that individuals divide up this correlation based on the number of connections they themselves possess [23]. This form of network autocorrelation model is known to result in somewhat downward-biased and conservative estimates of *ρ* [20], [24]–[26]. The downward bias, however, is substantial only at tie densities >0.15 [25], [26]. Our networks (see Results below) exhibited sufficiently low density that the concern of bias did not apply.

We used the Akaike Information Criterion (AIC) to compare the model fit of networks for predicting BMI. The AIC reflects the likelihood (probability of the data given the statistical model) after penalizing the model for the number of parameters. Lower AIC values reflect models that fit the data better, with differences >2 taken to indicate substantively different model quality.

### Comparing Network Measures

We calculated common network measures for each individual on the Name Generator Network and on each of the three email networks using the R package “igraph” [27]. We calculated all measures on the largest connected components from the networks. A connected component of a network is a set of individuals in which one can travel along social ties from any individual to any other. Disconnected components have gaps between them because there is not even one social tie between any individuals in the two different components. The largest connected component includes the greatest number of individuals compared to the other connected components of a network.

We calculated degree, betweenness, closeness, eigen vector centrality, and transitivity for each individual in the largest connected component of each email network. We extracted these same measures from the largest connected component of the Name Generator. The correspondence of each metric as calculated from email or survey data indicates a different aspect of how well the email networks replicated the survey network.

The network metrics can be described as follows. Readers interested in more detailed exposition on network metrics are recommended to Wasserman and Faust [16].

- *Degree*: the number of ties that an individual shares with others.
- *Betweenness*: the total number of shortest paths in the network that go through a given individual.
- *Shortest paths*: connections between all pairs of individuals that minimize the number of social ties one must cross to go from one individual to another.
- *Closeness centrality*: the average shortest path distance from an individual to all others.
- *Eigen vector centrality*: the distributed connectedness of an individual by assessing the extent to which an individual has many social ties to others who themselves have many social ties.
- *Transitivity*: the proportion of an individual’s social ties who are also tied to each other (i.e. are my friends also friends?).

We examined how the median degree and degree range compared across the various networks, again using only those individuals who appeared in the largest connected components of both the email networks and Name Generator Networks. We then assessed the associations of these measures between each of the email networks and the Name Generator Network by comparing each measure through a network autocorrelation model with the Name Generator measure set as the dependent variable and a given email network measure as the independent variable. In the case of transitivity, we eliminated any individuals who had an undefined value on either network in the comparison. Transitivity is undefined for individuals with degree of one. Because all six measures are nonindependent across individuals, we included Name Generator and the appropriate email row-normalized adjacency matrices to adjust the residuals. Thus, when testing egocentric measures from the Ranked Partner email network, we used the row-normalized Ranked Partner matrix and the row-normalized Name Generator matrix. When testing measures from the Single Recipient network, we used the row-normalized Single Recipient matrix, etc. In this case, we were not testing our main hypotheses through the network autocorrelation parameter, *ρ*, as these matrices only served to appropriately adjust the model residuals for nonindependence. The tests of the associations of egocentric network metrics were through the slope coefficient assessed for the regression of a specific Name Generator metric on each email network metric. We log transformed all egocentric metrics except for closeness centrality because visual inspection of their distributions revealed them to be right skewed. All Name Generator and email-based egocentric metrics were then converted to z-scores with mean of 0 and standard deviation of 1 such that they were all on the same scale. This was done to facilitate easier interpretation of the slope coefficients and to eliminate the complications introduced from having intercept terms being appropriate for some tests and not others. Because all metrics were standardized to a mean of zero, intercept terms were unnecessary.

## Results

### Description of Data Collected

Among 2468 Healthways employees, 1159 individuals responded to the Name Generator Survey. Respondents named 1198 individuals who themselves did not respond to the Name Generator, such that 2357 individuals were included in the offline social network due to their inclusion in the Name Generator data.

The five month email log from Healthways included records of 2,369,072 single recipient emails and 3,386,743 multiple recipient emails. In our analyses we used all emails among Healthways’ employees for which either the sender or recipient was included in the Name Generator Network. However, because only the sender or receiver was required to be in the offline data set, there were senders and recipients who were unique to the email networks. The individuals included in a given analysis thus depended on the threshold set for inclusion and which networks were being compared.

### Optimizing Email Networks to the Name Generator Survey

Each method of constructing networks from email data produced similar correlations with the Name Generator ties, which we assessed on the 1992 individuals present in both the Name Generator and Email networks (Table 3). Email networks exhibited higher density of ties than did the Name Generator Network. The Ranked Partner Network stood out the most distinctly in this regard (Table 3).

**Table 3:** Network tie densities and correlations of email networks with the Name Generator.

Email Network	Correlation with NameGenerator	Tie density (No. ties/No. undirected pairwise relations)*	Proportion Name Generator ties recovered
Single Recipient Network†	0.35	0.004	0.45
Logistic Regression Network‡	0.37	0.005	0.54
Ranked Partner Network§	0.36	0.006	0.54

N = 1992 individuals who were common to both the email and Name Generator networks.

* Tie density in the Name Generator Network equaled 0.003, N ties = 4989.

† average single recipient emails >2 per week.

‡ logistic regression fitted value >0.09.

§ single recipient emails rank > = 13.

Some individuals did not respond to the Name Generator but were named by others and thus occurred in the overall network. This introduced the possibility of spurious results of the analysis above (Table 3) from sparse sampling of the ties of nonrespondents to the Name Generator. To assess the robustness of these results to this sampling problem, we reevaluated the optimal thresholds for email networks by correlating to the Name Generator network using only those individuals who responded to the survey. This procedure reduced the sample size to 1001 individuals who responded to the survey and who were present in the email data. The analyses produced generally similar optimal thresholds as when all individuals were included. Table 3 shows the best threshold for the Single Recipient Network using all the data was exchanging more than 2 emails per week (correlation with Name Generator = 0.35), while when using only survey respondents the best threshold was exchanging more than 1.8 emails per week (correlation with Name Generator = 0.43). Comparing the Logistic Regression Networks, the optimal threshold with all the data was 0.09 predicted probability of a tie (correlation with Name Generator = 0.37), while with only the respondents included it was 0.06 (correlation with Name Generator = 0.45). The optimal threshold for the Rank Partner Network was identical when using either all the individuals or the respondents only (include all ranks 1 through 13), but using the survey respondents produced a higher correlation to the Name Generator (r = 0.45) than did using all the individuals (r = 0.36).

**Figure 1:**
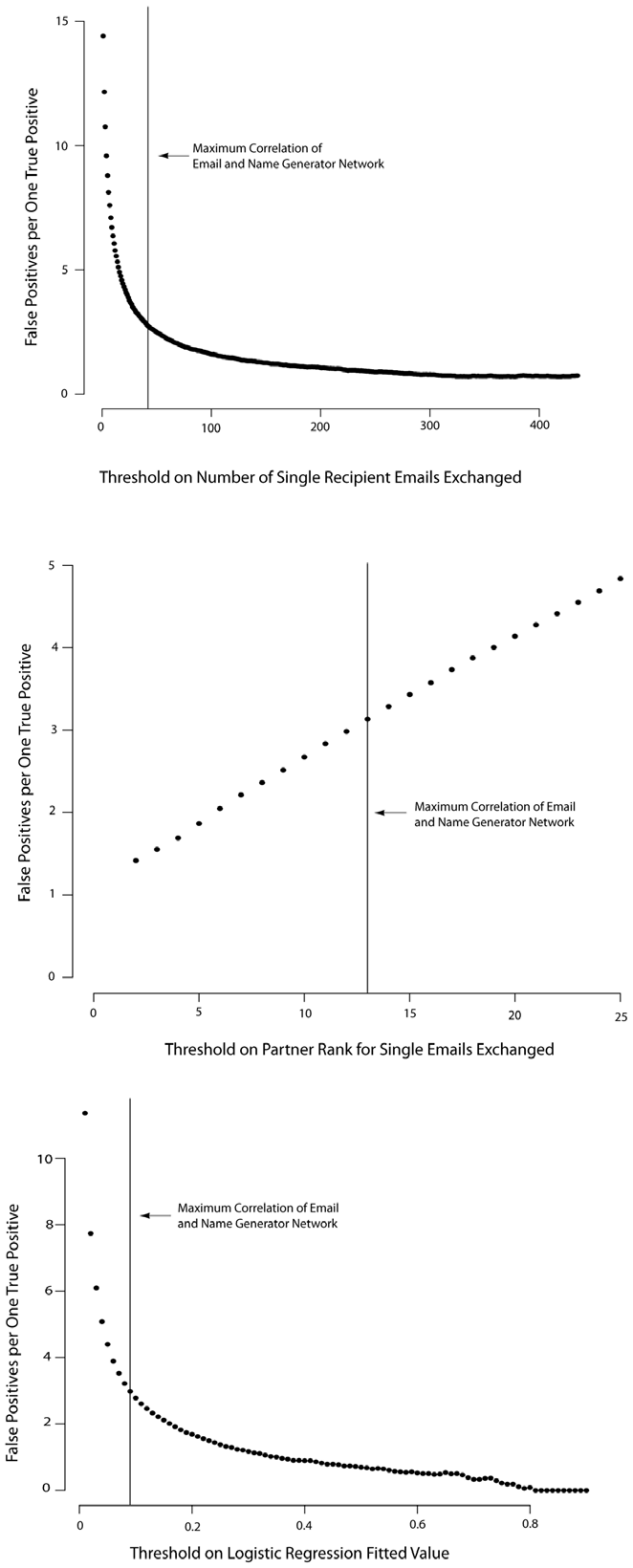
Ratios of false to true positives during threshold optimization of email networks on the Name Generator Survey network. Networks used in all subsequent analyses were derived from the thresholds that produced the highest correlations between the Name Generator and email networks (vertical lines).

**Figure 2:**
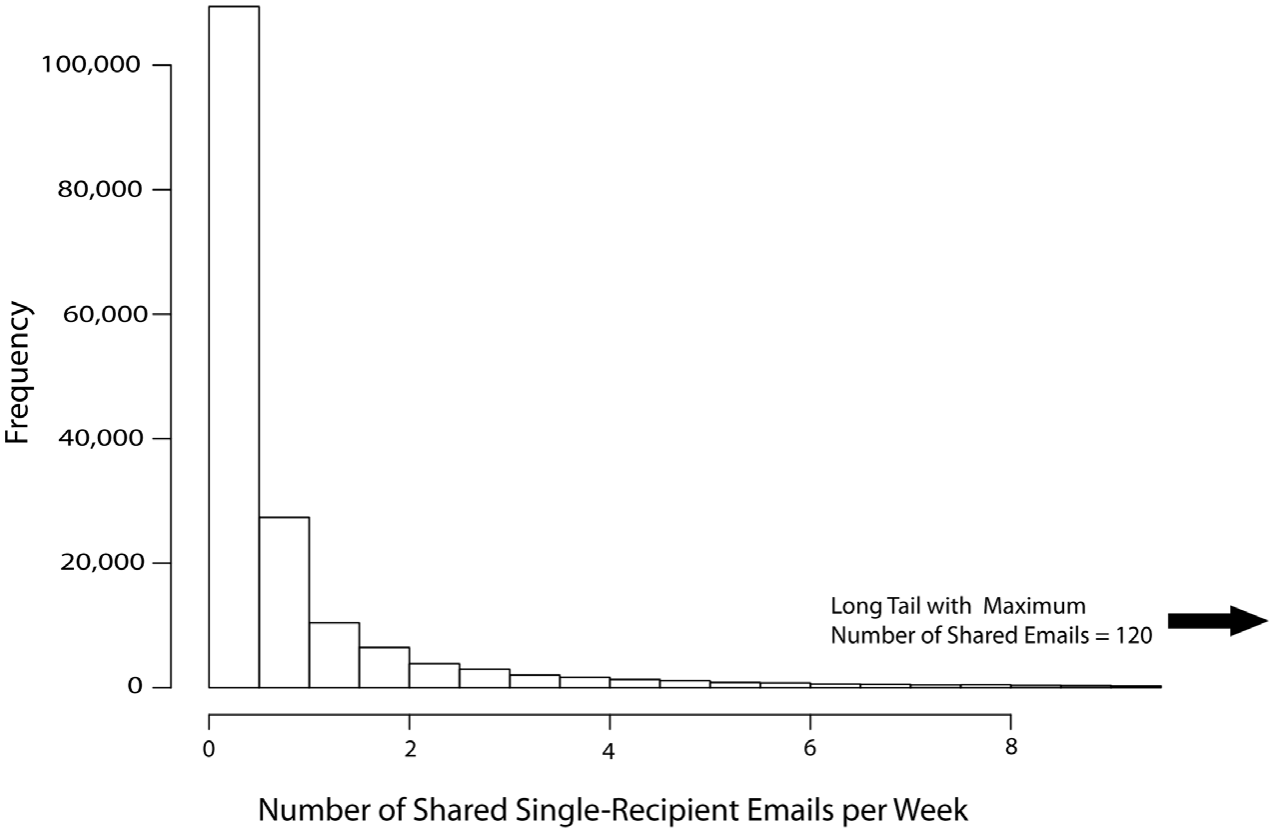
Distribution of the total emails exchanged between all pairs for the 5 month dataset used in the study.

We plotted the ratio of false:true positives obtained by our optimization of the correlation coefficients for each email network to the name generator network (Figure 1). For the purpose of generating these plots, an email tie constituted a true positive if it had a corresponding Name Generator tie. An email tie was a false positive if it had no corresponding Name Generator tie. The sample was not limited to survey respondents. The distribution for the unthresholded number of emails exchanged per week is shown as Figure 2.

False positives also can be assessed as a false positive rate by calculating the number of false positive results divided by the total pairs of individuals that do not have a Name Generator tie between them, regardless of whether the pair had an email tie. Although the false positive rates were very low in absolute value, the false:true positive ratio at the optimal correlation value (threshold) remained substantial (Figure 1). This occurred because the frequency of Name Generator ties is very sparse compared to the number of pairwise relations. In the Name Generator Network, there are 333 pairwise relations without a tie for every one relation with a tie, and any of these 333 relations could be predicted to be a tie by the email network. Thus, a low false positive rate (in all email networks <0.006) still resulted in a large number of false positive ties (social ties without corresponding offline ties, see also [13]). All the email networks inferred less than one false positive per 167 pairs of individuals who did not have a Name Generator tie between them. Because of the great number of pairs without Name Generator ties, this low false positive rate still produced networks with between two and four false positives for every true positive being inferred by the various email networks at the optimal threshold value (Figure 1).

### Testing Email Networks against Body Mass Index with Network Autocorrelation

BMI exhibited significant network autocorrelation on all four networks examined, with p-values for the z-statistic of *ρ* being <0.001 in all cases. This indicates that individuals have BMI values that are similar to their social connections. The data for the autocorrelation analysis included 1660 individuals who appeared in all three data sets: email logs, Name Generator Network, biometric BMI data. We used AIC criteria to evaluate how well each of these networks explained variation in BMI. AIC is derived from calculating the probability (likelihood) of the dependent variable’s observed distribution under a given network model. Lower AIC values reflect better model fit.

The Ranked Partner Network produced the best network for predicting BMI and the best model fit among those evaluated (AIC = 11091, *ρ* = 0.48). The autocorrelation of 0.48 indicated that about half of the deviation from the mean BMI is correlated across individuals tied by the Ranked Partner Network. The Ranked Partner Network model for BMI also had a substantially better AIC value than the Name Generator Network (AIC = 11108, *ρ* = 0.27). The Logistic Regression Network performed about equally well as the Name Generator Network for predicting BMI (AIC = 11107, *ρ* = 0.37), while the model statistics of the Single Recipient Network were worse than all other networks (AIC = 11142, *ρ* = 0.24).

As with the optimization of the email thresholds to the Name Generator network, we assessed the robustness of this result using the subset of individuals who themselves responded to the Name Generator survey. This was important for the BMI autocorrelation as this represents the one empirical check of our approach to building social networks against data (BMI) that is truly independent of the measurement of network ties. As with the optimization of email thresholds, we found that the same email network performed best using the subset of respondents, of whom 848 had also provided BMI and were present in the email logs. Specifically, as with the full data set, the Ranked Partner Network produced the lowest AIC value and the highest autocorrelation parameter among the email networks (AIC = 5671, *ρ* = 0.38). In contrast to the results with all the individuals, the Name Generator Network of respondents only had the best model fit (AIC = 5669, *ρ* = 0.30). The Logistic Regression Network had the same level of autocorrelation as did the Name Generator, but substantially worse model fit indicative of lower utility for predicting BMI (AIC = 5682, *ρ* = 0.30) As with the analyses that included all individuals, the Single Recipient Network was substantially worse at predicting BMI values (AIC = 5691, *ρ* = 0.26).

### Comparing Network Measures

We compared the match of common network measures on each of the email networks to the Name Generator Network. The median degree of the email networks was higher than the median degree of the Name Generator Network (Single Recipient = 8, Logistic Regression = 9, Ranked Partner = 14, Name Generator = 4).The range of degree values varied substantially across the different networks (Single Recipient = 1–70, Logistic Regression = 0–70, Ranked Partner = 1–171, Name Generator = 1–39).

We also assessed the associations of common egocentric network metrics via network autoregressive models (Table 4). The associations were measured by the estimated slope coefficients, which in this case effectively measure what proportion of a metric’s value as measured on an email network is also found in the same metric as measured on the Name Generator Network. The sample sizes for these comparisons varied because the largest connected components of the email networks varied, and egocentric network measures can be compared only across individuals in the same component. Sample sizes for all tests ranged from 1411–1608 (Table 4). Transitivity sample sizes were smaller than for the other network measures because transitivity is undefined for individuals with a single network connection.

**Table 4:** Slope coefficients from network autoregressive model with Name Generator metrics as the dependent variable (displayed with 95% confidence interval).

	Email Network		
Egocentric Metric	Single Recipient Network*	Logistic Regression Network†	Ranked Partner Network‡
Betweenness	0.32 (+−0.039)	0.34 (+−0.037)	0.25 (+−0.041)
Closeness	0.39 (+−0.041)	0.37 (+−0.041)	0.23 (+−0.044)
Transitivity	0.10 (+−0.051)	0.14 (+−0.053)	0.13 (+−0.058)
Eigenvector centrality	0.22 (+−0.039)	0.10 (+−0.053)	0.03 (+−0.062)
Degree	0.36 (+−0.039)	0.36 (+−0.036)	0.24 (+−0.033)

* N = 1839 except for Transitivity, which had N = 1411.

† N = 1922 except for Transitivity, which had N = 1574.

‡ N = 1951 except for Transitivity, which had N = 1609.

We found that email networks performed similarly for measures of betweenness, closeness, degree and transitivity (Table 4). The Ranked Partner Network exhibited lower associations on these measures than did the Single Recipient Network and Logistic Regression Network. None of the networks predicted transitivity well, although the confidence intervals for transitivity excluded 0 in all cases. One distinguishing feature of the eigenvector centrality from the Ranked Partner Network was that the confidence interval for its association with the same measure on the Name Generator Network did not exclude zero; thus, this measure on these two networks appears to be uncorrelated (Table 4). This likely indicates that substantially different sets of individuals have the highest tie density in the Ranked Partner network as compared to the Name Generator Network.

**Figure 3:**
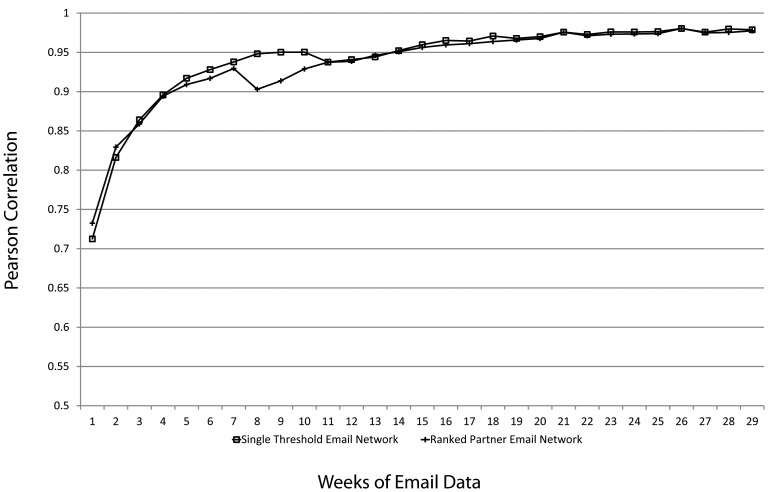
Stability of Single Threshold and Ranked Partner networks as additional weeks of email are added to the analysis. The y-axis shows the correlation of the network calculated from weeks 1 through N to the network calculated from weeks 1 through N+1.

Finally, we conducted a follow-up assessment of the robustness of these networks to the sample size of email collected. To do this, we recalculated these networks by adding one week increments starting with the first week and going out eight weeks beyond the data initially used in this study. The results (Figure 3) indicate that our five month sample (22 weeks) produced stable networks that would not have been substantially different had we sampled more weeks, or even if we had sampled several weeks fewer and stopped data collection after about 16 sampled weeks.

## Discussion

The results indicate that useful information about offline networks and traits can be extracted from networks generated by email data. These results confirm some findings from Wuchty and Uzzi (2011) [13]. These authors employed three methods of constructing networks from email data that were similar to our methods. Like our study, the previous researchers generated networks using three methods: (1) from a summation of emails in either direction for a pair, (2) a sum of emails sent and received normalized by volume per individual that resulted in directed ties (this is similar to our Ranked Partner method), and (3) a measure of reciprocation of email exchange for a pair (email reciprocity was represented in the scores for the asymmetry variable in our logistic regression method). As in our study, Wuchty and Uzzi (2011) found similar abilities of all these methods to predict survey ties in a sample of 31 managers from a single office for whom they had both survey and email data [13]. Our results confirm in much larger data set that various methods of inferring ties from emails perform similarly with respect to predicting survey-based ties and further extend upon this work by evaluating network effects with respect to a health trait.

Although the three alternative email networks exhibited similar levels of correlation to the survey-based ties in the Name Generator Network, they were quite different in their ability to predict an important health trait, BMI. Two transformations (multiple logistic regression and partner ranking) of email data into social networks of binary ties resulted in predictions of BMI that were as good or better than those provided by the name generator data. Although the Ranked Partner Network (generated by ranking single-recipient email partners) produced the best model for predicting BMI, its network metrics generally differed from the Name Generator more than the other two email networks –Single Recipient Network and Logistic Regression Network.

These results were robust to considering only the respondents to the Name Generator survey, rather than all the individuals who responded or were named, based on the fact that the same email network performed best at predicting BMI, the Ranked Partner Network. This robustness check was conducted to ensure that study results were not sensitive to potential confounding from a non-random group of employees that responded to the Name Generator survey who may have different traits than non-respondents who were named in that survey. The thresholds that maximized the correlation of email networks to the Name Generator network were similar for all three methods for constructing networks from email data. The correlations were all about 0.1 higher when using the respondents only rather can all the individuals, possibly because when including nonrespondents there were more missing ties in the Name Generator network that were present in the email networks, which would reduce the observed correlation between the two.

We found the Ranked Partner Network had the best AIC value and the highest level of autocorrelation, even outperforming the Name Generator Network for predicting BMI. The Logistic Regression Network exhibited similar predictive ability to the Name Generator Network as judged by comparison of the AIC values. In all networks, the estimated level of network autocorrelation was statistically significant, indicating that connected individuals have similar BMI values. Similar results were obtained through our analysis of respondents only, although the observed network autocorrelations were lower for the Ranked Partner and Logistic Regression networks. Using the respondents only, the Ranked Partner Network slightly underperformed the Name Generator network, as the AIC difference between them is 2, which is on the cusp of a meaningful difference in predictive value. This is consistent with the possibility that network ties were missing for the data set that included nonrespondents who were named in the survey, as the resultant Name Generator Network would fail to accurately model pathways of autocorrelation in BMI. This finding strongly supports the value of using email communications in an applied health intervention, because survey data are always much more incomplete when compared to the comprehensive records of employee email communications.

To our knowledge, this study is the first demonstration that physiological health traits covary along coworker ties inferred from either survey or email data [28]. Although the correlation of BMI across ties of the coworker network alone does not establish whether the cause is influence or homophily, if there were no correlation on coworker networks then social influence would be ruled out de facto. Additionally, population tendencies toward homophily in conjunction with social influence may actually create a resistance to change toward healthier habits if a group of connected individuals are not motivated to make a collective change. We would anticipate that traits more closely related to the work environment, such as worker perceptions of the company’s goals, progress on projects, or valued leaders, may covary even more strongly than health traits on a network derived from email data [29]. Future work should evaluate the extent to which other variables, such as those named or other health-related traits or behaviors, are predicted by network ties.

How can an email network outperform self-reports of friendship and health-relevant social ties? A full answer would entail an additional analysis that is beyond the aims of this study, but two differences between the email networks and the Name Generator Network are notable. First, the Ranked Partner Network has the highest density of ties (Table 3). Because our Name Generator Network included individuals who were named but did not respond, it likely underestimates the number of ties for these individuals. Second, the Ranked Partner Network may correlate more strongly than the Name Generator with some unmeasured confounding variable. From the standpoint of its utility solely as a predictive tool, the correlation of the Ranked Partner Network with some unmeasured confounder is not a disadvantage.

Not all network research, however, is aimed at uncovering patterns of trait correlation across ties. Network researchers often desire instead to establish the structural positions of individuals in a network (e.g. are they central or peripheral in the network). Regarding structural positions, the Single Recipient Network and Logistic Regression Network exhibited moderate associations for closeness, betweenness, and degree centrality relative to the Name Generator Network. We found the egocentric measures from the Ranked Partner Network generally matched significantly less well to the Name Generator than did the other email networks (Table 4). The fact that its confidence interval for eigenvector centrality encompassed zero may indicate that the Ranked Partner Network exhibits fundamental differences in the areas of the network with greatest tie density.

Overall, the Ranked Partner and Single Threshold networks performed best, but for different purposes. Using the rank transformation of email data to social network ties, we obtained substantial explanatory power for a trait, BMI, that was completely independent of how we optimized the thresholding on email ranks. This finding may indicate that the correct specification of email networks can replace or even surpass the use of surveys for understanding trait covariation in networks. The Ranked Partner Network, however, would not be ideal for tasks that focused on the structural positions of individuals within networks. For example, researchers who are interested in the power dynamics of individuals in a network would want to accurately capture eigenvector centralities (an individual’s level of connection to well-connected individuals). Our research indicates that a threshold of 2 single recipient emails per week would be best at recovering eigenvector centrality and many other network metrics. The findings presented herein provide a solid empirical basis for selecting the correct strategy to infer a social network from email data given a set of research goals.

A limitation of this work is the data are necessarily a sample of the Healthways population who either responded to or were named in the Name Generator Survey. Additionally, Healthways represents only one corporate setting, and findings at this location may or may not generalize to other corporate employers. In conclusion, the results presented demonstrate that email data can be used to map social networks within an employer’s population and that meaningful correlations of BMI occur in each network across network connections among coworkers. Email provided an advantage over survey-derived data because survey-derived networks are typically incomplete due to low response rates, whereas email data can be obtained for all members of a population. Inferring social networks using email data via one of the approaches tested presents a promising approach for employers or other organizations to better understand their population with respect to BMI patterns, and future work should aim to elucidate how email-derived network maps could be used to impact health in these populations.
